# Impaired ATP hydrolysis in blood plasma contributes to age-related neutrophil dysfunction

**DOI:** 10.1186/s12979-024-00441-4

**Published:** 2024-07-03

**Authors:** Carola Ledderose, Eleftheria-Angeliki Valsami, Mark Elevado, Qing Liu, Brennan Giva, Julian Curatolo, Joshua Delfin, Reem Abutabikh, Wolfgang G. Junger

**Affiliations:** 1 Department of Surgery, University of California, San Diego Health, 9452 Medical Ctr Dr, La Jolla, San Diego, CA 92037 USA; 2 Department of Surgery, Beth Israel Deaconess Medical Center, Harvard Medical School, Boston, MA USA

**Keywords:** Aging, Mice, Neutrophil dysfunction, Purinergic signaling, ATP hydrolysis

## Abstract

**Background:**

The function of polymorphonuclear neutrophils (PMNs) decreases with age, which results in infectious and inflammatory complications in older individuals. The underlying causes are not fully understood. ATP release and autocrine stimulation of purinergic receptors help PMNs combat microbial invaders. Excessive extracellular ATP interferes with these mechanisms and promotes inflammatory PMN responses. Here, we studied whether dysregulated purinergic signaling in PMNs contributes to their dysfunction in older individuals.

**Results:**

Bacterial infection of C57BL/6 mice resulted in exaggerated PMN activation that was significantly greater in old mice (64 weeks) than in young animals (10 weeks). In contrast to young animals, old mice were unable to prevent the systemic spread of bacteria, resulting in lethal sepsis and significantly greater mortality in old mice than in their younger counterparts. We found that the ATP levels in the plasma of mice increased with age and that, along with the extracellular accumulation of ATP, the PMNs of old mice became increasingly primed. Stimulation of the formyl peptide receptors of those primed PMNs triggered inflammatory responses that were significantly more pronounced in old mice than in young animals. However, bacterial phagocytosis and killing by PMNs of old mice were significantly lower than that of young mice. These age-dependent PMN dysfunctions correlated with a decrease in the enzymatic activity of plasma ATPases that convert extracellular ATP to adenosine. ATPases depend on divalent metal ions, including Ca^2+^, Mg^2+^, and Zn^2+^, and we found that depletion of these ions blocked the hydrolysis of ATP and the formation of adenosine in human blood, resulting in ATP accumulation and dysregulation of PMN functions equivalent to those observed in response to aging.

**Conclusions:**

Our findings suggest that impaired hydrolysis of plasma ATP dysregulates PMN function in older individuals. We conclude that strategies aimed at restoring plasma ATPase activity may offer novel therapeutic opportunities to reduce immune dysfunction, inflammation, and infectious complications in older patients.

## Background

Aging is an integral aspect of mammalian life. Advances in medicine have extended human life expectancy, and the average lifespan of humans is now 73 years, which is approximately 10 years longer than that in 1990. According to a United Nations report, 20% of the people in Europe and North America and approximately 10% worldwide are over 65 years old. However, dysregulated immune responses cause inflammatory and infectious complications that greatly diminish the quality of life among aging individuals [1–3].

A contributing factor is the impairment of polymorphonuclear neutrophils (PMNs) in older people, which results in excessive inflammation and impaired antimicrobial defenses [4–6]. PMNs are the most abundant leukocyte subtype in human blood. Their primary task is to fend off microbial invaders. PMNs possess highly efficient mechanisms for recognizing, pursuing, and killing microorganisms with a powerful arsenal of cytotoxic mediators [7]. For that purpose, PMNs possess a variety of specialized receptors that recognize bacteria. These include formyl peptide receptors (FPRs), which are sensitive detectors of formylated peptides that are released from bacteria and damaged mitochondria [8]. Stimulated PMNs move to affected host tissues, where they assume a polarized cell shape that allows them to infiltrate sites of infection [9]. This ability of PMNs to detect and target microbial invaders involves intricate autocrine signaling mechanisms that include the release of cellular ATP and the stimulation of purinergic receptors that are redistributed across the cell surface of PMNs [10].

All mammalian cells depend on ATP as an intracellular energy carrier that fuels cell functions. However, mammalian cells, including PMNs, also actively release ATP into the extracellular space, where ATP acts as an autocrine signaling molecule that orchestrates these cell functions [10, 11]. Extracellular ATP and its breakdown products stimulate purinergic receptors, which comprise three families, namely, the P1, P2Y, and P2X receptors. All seven P2X receptor subtypes and some of the eight P2Y receptors recognize ATP, while the ATP breakdown product adenosine is the ligand of the four P1 receptors [12]. Among these different purinergic receptors, PMNs express primarily the A2a and P2Y2 receptor subtypes. FPR stimulation causes rapid release of ATP via pannexin-1 channels, resulting in the stimulation of nearby P2Y2 receptors that amplify FPR signaling and induce cell activation [13]. Extracellular ATP is subsequently converted to ADP, AMP, and adenosine, which can be internalized via specific transporters and recycled to replenish intracellular ATP levels [14]. Rapid removal of extracellular ATP and adenosine is essential for preventing PMN dysregulation because uncoordinated stimulation of purinergic receptors disrupts the autocrine signaling mechanisms that regulate PMN functions [15]. Under physiological circumstances, stimulation of P2Y2 and A2a receptors occurs in a coordinated fashion at the front and back of cells to facilitate the formation and stabilization of a polarized cell shape [16, 17].

Increased extracellular ATP levels in response to trauma, inflammation, and sepsis disrupt the modulatory actions of the P2Y2 and A2a receptors of PMNs, which results in deranged chemotaxis and impaired antimicrobial host defenses [18]. Extracellular ATP accumulation promotes PMN dysfunction and the release of cytotoxic mediators that subsequently damage host tissues [19]. PMN dysfunction and host tissue damage increase the severity of complications among intensive care patients following traumatic injuries, bacterial or viral infections, and sepsis [20–23].

The risk of death among these patients sharply increases with age [24–26]. Several studies have demonstrated that aging leads to defects in PMN functions that impair host defenses [4, 5, 27]. However, detailed information about the underlying mechanisms is still lacking [6]. The aim of this study was to examine whether aging alters the purinergic signaling mechanisms of PMNs and how such changes contribute to the dysregulation of this important immune cell population.

## Methods

### Mice

The Institutional Animal Care and Use Committees of Beth Israel Deaconess Medical Center and of the University of California San Diego approved all animal experiments, which followed the guidelines for the Care and Use of Laboratory Animals of the National Institutes of Health. C57BL/6 mice (Jackson Laboratory, Bar Harbor, ME) were housed in groups of two to five mice per cage with free access to standard rodent food and water. Male and female mice ranging in age from 8 to 81 weeks were used for the different experiments.

### Cecal slurry infection model

For the preparation of the fecal slurry, fresh cecal contents of healthy euthanized donor mice were adjusted to 100 mg/ml in a solution of sterile saline and 15% glycerol and stored in aliquots at -80 °C to ensure standardized conditions of infection throughout the study. Male and female mice of different ages were subjected to intraperitoneal injections of cecal slurry (0.1–0.8 mg/g body weight). Survival and body temperature were monitored for up to 48 h. Five physiological parameters (posture, appearance, spontaneous activity, responsiveness, and respiration rate and quality) were assessed at least every 2 h, and each parameter was scored on a scale from 0 (normal) to 4 (severely impaired). The five parameters were combined into a single sepsis score ranging from 0 to 20. Animals were euthanized when the body temperature fell below 30 °C, when the respiration was scored 3 or 4, or when any other parameter reached a score of 4.

### Bacterial counts

Serial dilutions of blood samples were plated on LB agar plates (MP Biomedicals, Solon, OH). Colony-forming units (CFU) were counted after incubation at 37 °C for 18 h.

### PMN activation assays

Whole blood samples were obtained from the mice via thoracotomy and cardiac puncture under deep isoflurane anesthesia using heparinized syringes. Heparinized blood was immediately stained with the following antibodies (BioLegend, San Diego, CA): anti-CD11b APC (clone M1/70), anti-Ly6G Brilliant Violet 421 (clone 1A8), anti-CD62L FITC (clone MEL-14), and anti-CD63 PE (clone NVG-2). In some cases, functional PMN responses were assessed following stimulation with the FPR agonist WKYMVm (W-peptide; Tocris Bioscience, Minneapolis, MN) as previously described [28]. Briefly, blood samples were warmed to 37 °C and stimulated with 50 nM W-peptide for 15 min to assess the surface expression of the activation markers CD11b and L-selectin (CD62L), or they were stimulated with 1 µM W-peptide for 5 min to analyze the expression of the degranulation marker CD63. For the assessment of reactive oxygen species (ROS) production, blood samples were stained with 100 µM dihydrorhodamine-123 (DHR; Invitrogen, Carlsbad, CA) at 37 °C and stimulated with 100 nM W-peptide for 20 min. In some experiments, ATP (0.01–100 µM; Sigma-Aldrich, St. Louis, MO) was added at the time of stimulation. The samples were treated with RBC lysis/fixation buffer (BioLegend, San Diego, CA) and analyzed with a NovoCyte 3000 flow cytometer (Agilent, Santa Clara, CA). PMNs were identified as CD11b + Ly6G + cells. Fluorescence-minus-one controls were used to define positive cell populations.

### Phagocytosis and bacterial killing assays

Whole blood samples were mixed 1:1 with Hanks’ balanced salt solution (HBSS; HyClone Laboratories, Logan, UT) and incubated with 100 µg/ml pHrodo Green *E. coli* bioparticles (Invitrogen, Thermo Fisher Scientific, Waltham, MA) for 30 min at 37 °C. Reactions were stopped on ice, PMNs were labeled with anti-CD11b and anti-Ly6G antibodies, and the percentages of PMNs engaged in phagocytosis and their mean fluorescence intensities (MFIs) were recorded as readouts of phagocytosis capacity using flow cytometry. Bacterial killing was assessed by incubating 100 µl blood samples from young adult mice (12 weeks) with 2,000 CFU *E. coli* DH5α in the presence of different concentrations of ATP at 37 °C. After 2 h, the remaining bacteria were counted on LB agar plates as described above.

### Functions of human PMNs

All experiments involving blood from healthy human adults (25–42 years old) were approved by the Institutional Review Boards of Beth Israel Deaconess Medical Center and the University of California San Diego, and written informed consent was obtained prior to blood collection. Heparinized blood samples were incubated with different concentrations of ethylenediaminetetraacetic acid (EDTA; 0.1-5 mM; Sigma‒Aldrich, St. Louis, MO) at 37 °C for 2 h. To analyze the effect of EDTA treatment on ROS production, blood samples were then stained with DHR (100 µM) and stimulated with 50 nM fMLP (Sigma‒Aldrich, St. Louis, MO) at 37 °C for 20 min. Degranulation was assessed by stimulating cells for 5 min with 15 nM fMLP followed by staining with anti-human CD63 Brilliant Violet 421 antibodies (clone H5C6; BioLegend, San Diego, CA) on ice. The samples were treated with RBC lysis/fixation buffer and analyzed by flow cytometry. Human PMNs were identified based on their characteristic FSC/SSC properties.

### ATP breakdown

To assess the ATP breakdown capacity of mouse or human plasma, heparin plasma samples were diluted 1:4 with HBSS, warmed to 37 °C, and mixed with 2 µM ATP (Sigma‒Aldrich, St. Louis, MO). The reactions were stopped on ice at the indicated time points by the addition of 800 mM perchloric acid (Sigma‒Aldrich, St. Louis, MO). In some experiments, ATP breakdown in heparinized blood samples was analyzed after the addition of EDTA (0.05-5 mM). The concentrations of ATP, ADP, AMP, and adenosine were determined via high-performance liquid chromatography (HPLC) as described below.

### Analysis of ATP and ATP breakdown products by HPLC

Plasma ATP concentrations were determined as previously described [29]. Briefly, plasma was prepared from chilled heparinized blood samples, stabilized with 800 mM perchloric acid, and spiked with 250 nM of the internal standard adenosine 5’-(α,β-methylene)-diphosphate (Sigma‒Aldrich, St. Louis, MO). Fluorescent 1,N^6^-ethenoderivatives of adenine compounds were generated, pre-purified by solid phase extraction, concentrated, and analyzed with an Agilent 1260 Infinity HPLC system (Agilent, Santa Clara, CA). Plasma concentrations of ATP, ADP, AMP, and adenosine were calculated based on a 500 nM standard mixture of these compounds that was processed and analyzed in parallel.

### Determination of role of Zn^2+^ in plasma ATPase activity

Heparinized human blood samples were incubated with increasing concentrations of EDTA (0.05-5 mM) for 2 h. Then, plasma was collected, and ATP, adenosine, Ca^2+^, and Mg^2+^ concentrations were determined using HPLC as described, and Ca^2+^ (Cayman Chemical, Ann Arbor, Michigan) and Mg^2+^ (Sigma‒Aldrich, St. Louis, MO) assay kits as instructed by the manufacturers. The role of Zn^2+^ as a coenzyme of ATPases in mouse plasma was assessed by depleting plasma samples of divalent ions by the addition of 100 µM EDTA. Plasma was diluted with HBSS without Ca^2+^ and Mg^2+^ (HBSS w/o Ca Mg) at a ratio of 1:50 and incubated with 100 µM EDTA in HBSS w/o Ca Mg for 10 min at 37 °C. Then, increasing concentrations of ZnCl_2_ (also dissolved in HBSS w/o Ca Mg) were added and samples were incubated for another 10 min at 37 °C. Then, ATP was added to each sample at a final concentration of 1,000 nM and the ATP remaining after a 30-min incubation period at 37 °C was determined with a luciferase assay kit (CellTiter-Glo® 3D Cell Viability Assay; Promega, Madison, WI).

### Statistical analyses

Unless otherwise stated, the results are reported as the mean ± standard error of the mean (SEM) of at least 3 independent experiments per group. Differences between groups were tested for statistical significance using unpaired two-tailed t tests for comparisons between two groups and one-way ANOVA followed by the Holm-Sidak test for comparisons among multiple groups. Survival data were plotted as Kaplan-Meier curves, and groups were compared with the log-rank Mantel-Cox test. Pearson’s correlation analysis was used to test for correlations. Differences were considered statistically significant at *p* < 0.05. All the statistical analyses were performed using GraphPad Prism software (San Diego, CA).

## Results

### Plasma ATP levels increase with age

**Fig. 1:**
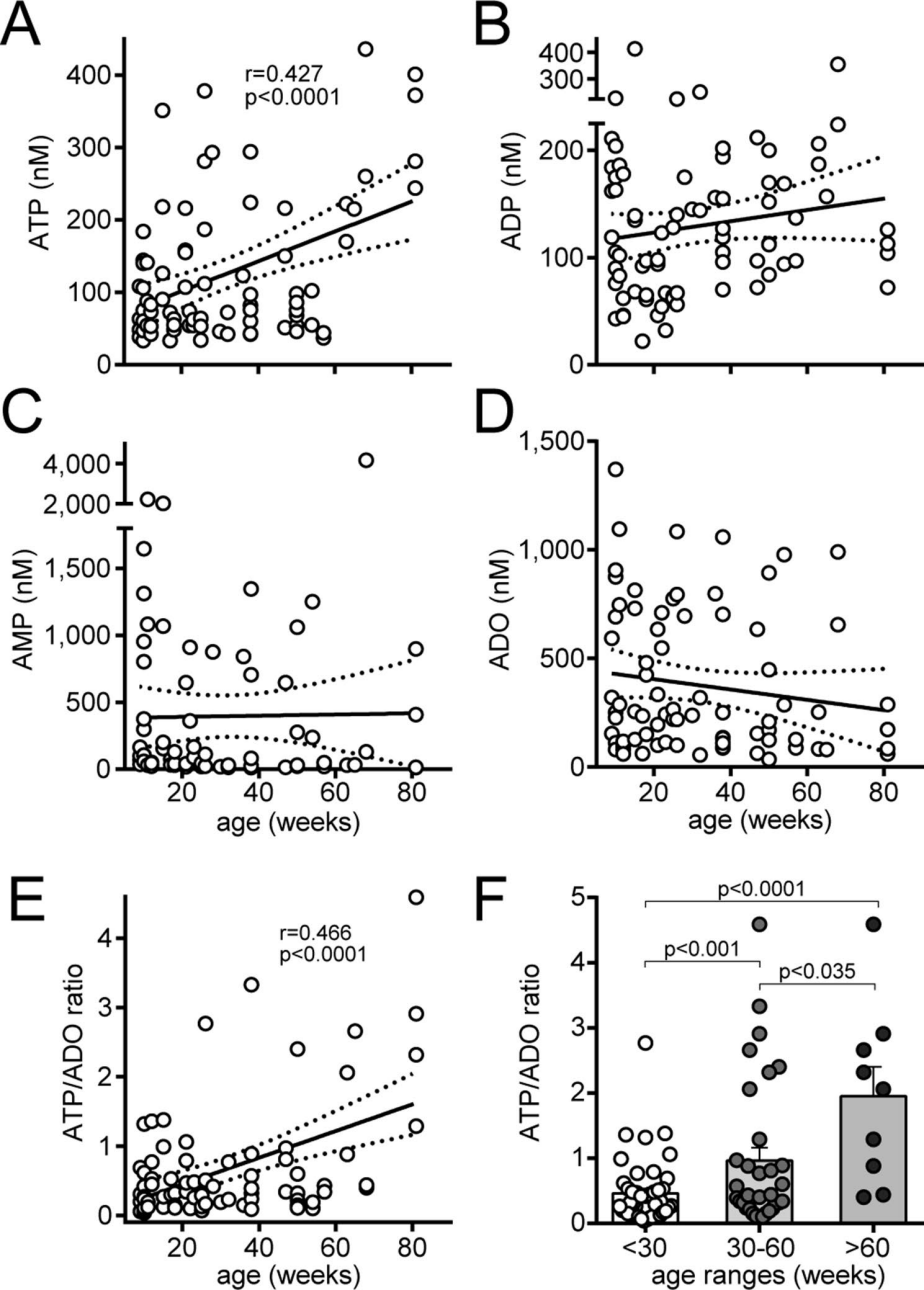
ATP concentrations in blood plasma increase with age. The concentrations of ATP and its breakdown products ADP, AMP, and adenosine (ADO) in the plasma of healthy mice of different ages were assessed via HPLC analysis (**A-D**). The ratio of the plasma ATP concentration to the ADO concentration in the mice was compared with age (**E-F**). Linear correlations with 95% confidence intervals are shown along with correlation coefficients in panels A and E. Data in panel F are shown as the mean values ± SEMs, and age groups were compared with one-way ANOVA and Holm-Sidak post hoc test

Aging is accompanied by impairments of PMN function, which results in increased susceptibility to infectious and septic complications [6, 25]. Our previous studies have shown that the accumulation of ATP in the extracellular environment also impairs PMN function [18, 19, 30–32]. Therefore, we wondered whether plasma ATP levels increase with age and whether this increase contributes to PMN dysfunction in older individuals. We evaluated this hypothesis using C57BL/6 mice ranging in age from 8 to 81 weeks. We collected blood samples from these animals and assessed their plasma levels of ATP and its breakdown products by HPLC analysis. Plasma ATP concentrations indeed significantly increased with age (Fig. 1A). Although ADP, AMP, and adenosine levels were not correlated with age (Fig. 1B-D), the ratio between the concentrations of ATP and its breakdown product adenosine did and increased significantly in response to aging (Fig. 1E-F).

### Old mice are more susceptible to lethal sepsis than young mice

**Fig. 2:**
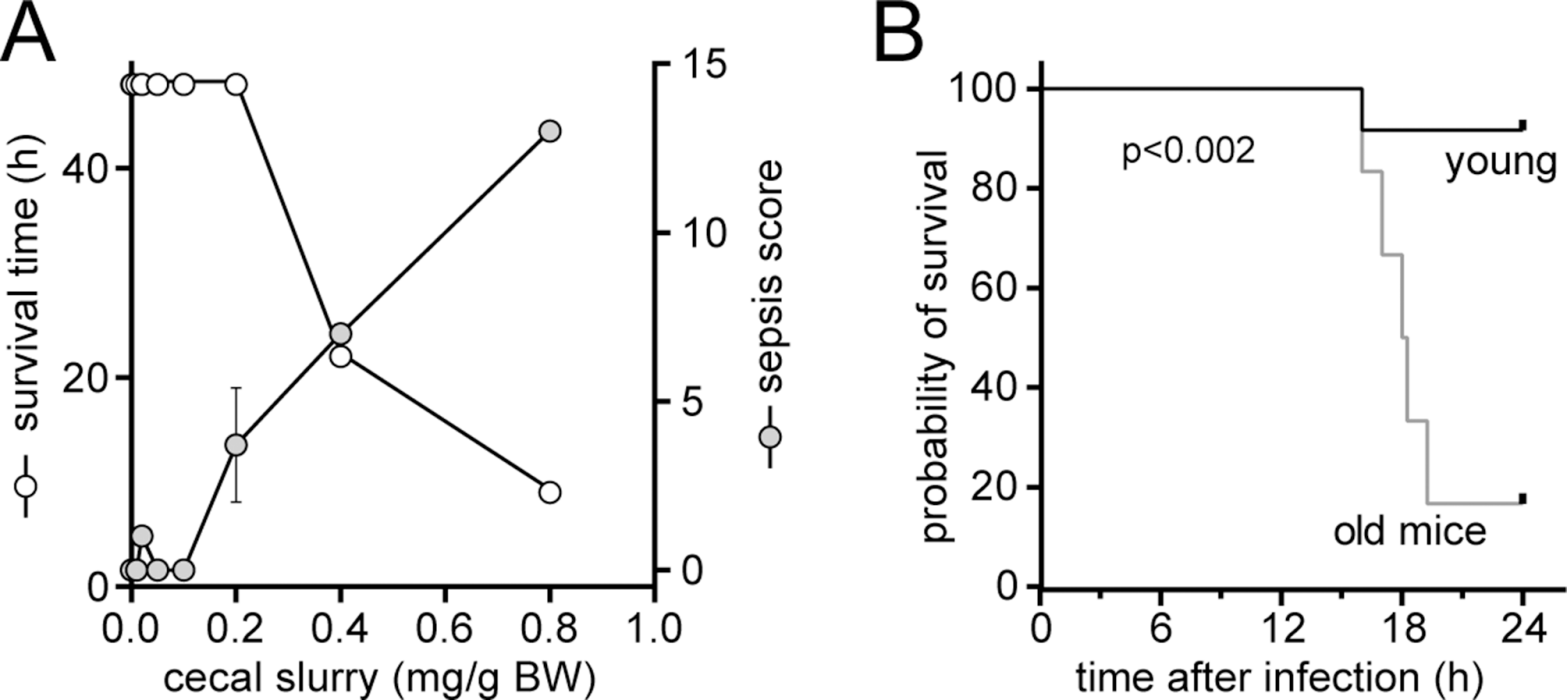
Polymicrobial infection causes lethal sepsis in old but not young mice. C57BL/6 mice ranging in age from 8–19 weeks (*n* = 13) were subjected to polymicrobial infection by intraperitoneal injection of different doses of a slurry of the cecal contents obtained from healthy adult mice. Survival was monitored for 48 h, and signs of sepsis were scored on a scale from 0–20 after 16 h (**A**). Young (10 weeks; *n* = 12) and old (64 weeks; *n* = 6) mice were subjected to equal doses of cecal slurry (0.3 mg/g body weight; BW), and clinical parameters and mortality were monitored at least every 2 h after injection for up to 24 h. The mortality of old versus young mice was compared with the log-rank Mantel–Cox test, *p* = 0.0014 (**B**)

Next, we studied the response of mice of different ages to intraabdominal infection. We established a sublethal polymicrobial infection model by challenging mice with peritoneal injections of different doses of pooled cecal slurry obtained from healthy adult mice. Doses of up to 0.2 mg/g bodyweight caused only slight symptoms of infection in mice ranging in age from 8 to 19 weeks. These animals recovered quickly, and no deaths occurred during the 48-hour observation period (Fig. 2A). Next, we challenged 10-week-old (*n* = 12) and 64-week-old (*n* = 6) mice with 0.3 mg/g bodyweight of the same cecal slurry preparation. This resulted in a single death among the young mouse population, whereas the mortality rate among the older mice was significantly higher (Fig. 2B). Unlike young mice, old mice developed severe signs of sepsis, which included a sustained drop in body temperature, an increase in the sepsis score, and elevated bacterial counts in the peripheral circulation that were 40 times greater than those of young mice (Table 1). These findings demonstrate that the risk of sepsis and death following infections is significantly higher in old mice than in young animals.

**Table 1:** Age differences in the response to microbial infection

	Healthy controls	Young infected	Old infected
Age (weeks)	40 ± 9	10	64
Bacteria (10^6^xCFU/ml blood)	0	4.0 ± 3.7	154.8 ± 97.4*
Body temperature (°C)	36.9 ± 0.2	36.0 ± 0.9	31.7 ± 1.1**
Sepsis score (0–20)	0	2 ± 1	9 ± 2**

Young (*n* = 12) and old (*n* = 6) mice were intraperitoneally infected with cecal slurry from healthy mice (0.3 mg/g BW). Posture, appearance, spontaneous activity, responsiveness, and respiration rate and quality were each scored on a scale from 0 (normal) to 4 (severely impaired) and combined into one sepsis score (0–20). Body temperatures and sepsis scores were determined 16 h after infection and compared to those of healthy animals (*n* = 35) that ranged in age from 9–81 weeks. Blood bacterial counts were measured at the time of euthanasia (16–24 h after infection). CFU; colony forming units. The data shown are the mean values ± SEMs. The results of old and young infected mice were compared with t tests; *p < 0.05, **p < 0.01

### Polymicrobial infections exacerbate PMN activation in old but not young mice

**Fig. 3:**
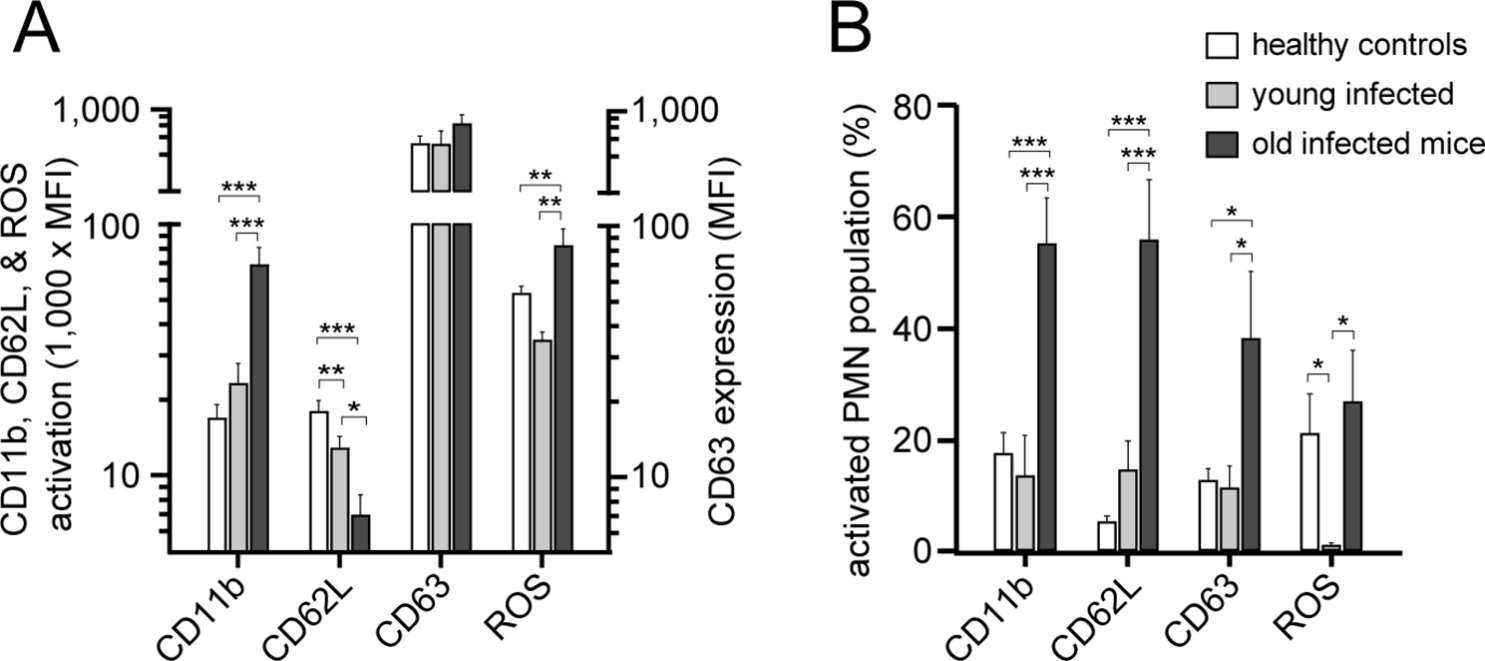
PMNs from old but not young mice respond to infection with excessive cell activation. Old (64 weeks; *n* = 6) and young (10 weeks; *n* = 6) mice were subjected to polymicrobial infections by intraperitoneal injection of cecal slurry (0.3 mg/g BW), and PMN activation levels were assessed by measuring CD11b, CD63, and CD62L (L-selectin) expression levels and the production of reactive oxygen species (ROS) 16 h after infection. Untreated animals (*n* = 15) with an average age of 40 ± 9 weeks (mean ± SEM) served as healthy controls. The degree of PMN activation (**A**) and the percentage of activated PMNs (**B**) were determined, and the results (shown as the mean values ± SEMs) were compared using one-way ANOVA and Holm-Sidak post hoc test; *p < 0.05, **p < 0.001, ***p < 0.0001. MFI: mean fluorescence intensity

An overwhelming inflammatory response of PMNs following infections can cause sepsis and host tissue damage [21]. Therefore, we studied the effect of the infectious challenge described above on the PMNs of old and young mice. We found that PMN activation responses 16 h after infection were significantly more pronounced in old animals than in young mice (Fig. 3). PMNs from old mice expressed significantly higher levels of the activation marker CD11b, showed increased shedding of CD62L (L-selectin), and produced more reactive oxygen species (ROS) than did PMNs from young, infected mice (Fig. 3A). In addition, a greater percentage of PMNs were activated in old than in young animals and expressed the degranulation marker CD63 (Fig. 3B). Infection of young mice did not elicit these PMN activation responses. Most PMN activation parameters tested in young mice did not differ from those of healthy controls (Fig. 3). These findings demonstrate that microbial infections result in strong PMN activation in old mice. The pathological consequences of excessive PMN activation include a predisposition to sepsis and high mortality in old mice after bacterial infection as shown above (Fig. 2B; Table 1).

### Extracellular ATP dose-dependently impairs PMN functions

**Fig. 4:**
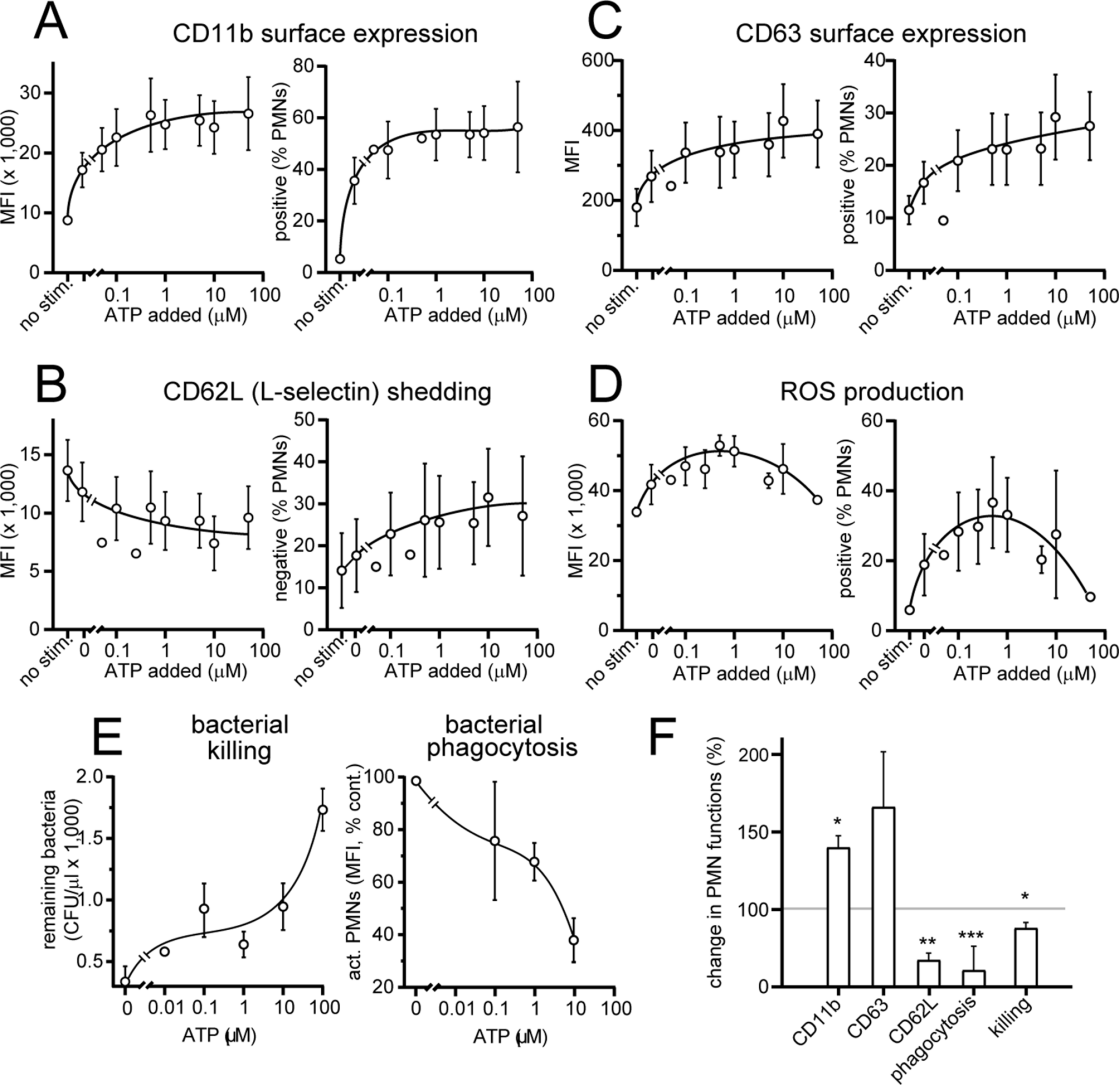
Extracellular ATP distorts the functional responses of mouse PMNs. The effects of adding increasing ATP concentrations to blood samples of healthy mice (12–16 weeks old) was assessed by stimulating PMNs with 50 nM (**A**, **B**) or 100 nM (**C**, **D**) W-peptide and determining CD11b expression (**A**), shedding of CD62L (**B**), expression of CD63 (**C**), production of ROS (**D**), and phagocytosis of fluorescence-labeled bacterial particles with flow cytometry, or by counting remaining live bacteria after their incubation with blood samples (**E**). The effects of ATP on CD11b and CD63 expression, CD62L shedding, bacterial phagocytosis, and killing of bacteria by mouse PMNs was assessed by comparing the responses of these cells before and after the addition of 10 µM ATP (**E**). The data shown are the means ± SEMs (A-C: *n* = 5, D: *n* = 3 E = 5 mice), and the data were compared with the corresponding control values (no ATP) using unpaired t tests; *p < 0.05, **p < 0.01, ***p < 0.001. MFI: mean fluorescence intensity

The results above suggest that the accumulation of ATP in the plasma of old mice may weaken the antimicrobial effectiveness of PMNs and elicit exaggerated inflammatory PMN responses that contribute to tissue damage and sepsis. Therefore, we examined how extracellular ATP levels affect the PMN responses of mice. We found that the addition of ATP to blood samples from healthy adult mice (< 20 weeks old) dose-dependently increased the expression of the activation marker CD11b (Fig. 4A), the shedding of CD62L (Fig. 4B), the expression of CD63 (Fig. 4C), and the production of ROS in response to FPR stimulation (Fig. 4D). The addition of ATP not only enhanced these inflammatory PMN responses but also impaired the phagocytosis and killing of bacteria by mouse PMNs (Fig. 4E-F), which suggests that ATP accumulation in response to aging impairs PMN-mediated host immune defenses.

### PMN priming and activation increase with age

**Fig. 5:**
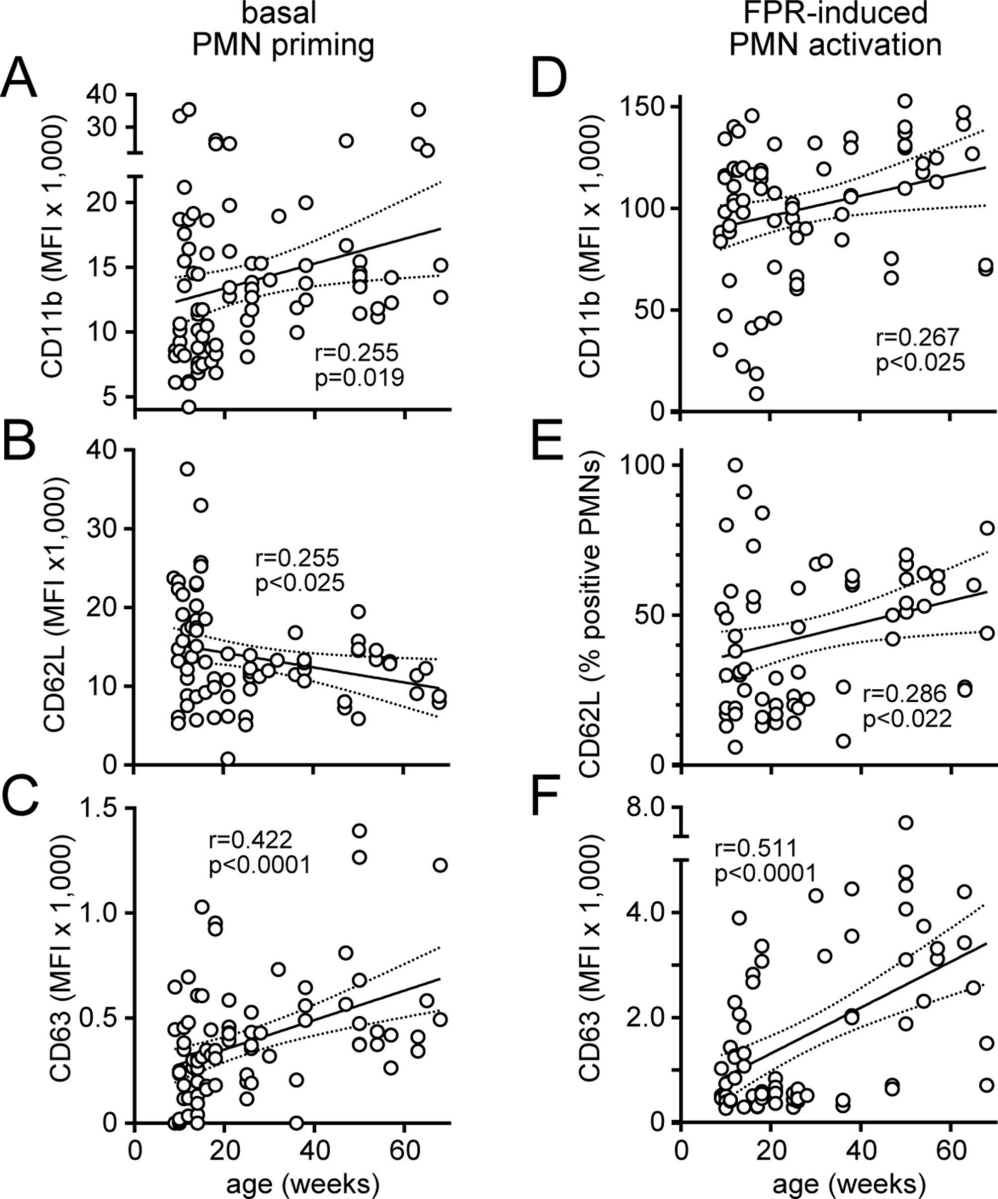
Priming and activation of PMNs increase with age. The basal levels of CD11b expression (**A**), CD62L shedding (**B**), and CD63 expression (**C**) on the surface of unstimulated PMNs in blood samples drawn from mice of different ages were determined by flow cytometry. Blood samples were stimulated with the FPR agonist W-peptide, and increases in CD11b expression (**D**), CD62 shedding (**E**), and CD63 expression (**F**) were assessed. Linear correlations with 95% confidence intervals are shown along with the corresponding correlation coefficients and significant p values. MFI: mean fluorescence intensity

By acting on P2Y2 receptors, ATP promotes the functional responses of PMNs [10, 33]. Excessive ATP accumulation in the plasma may therefore exacerbate inflammatory PMN responses in old mice. To test this hypothesis, we assessed PMN activation levels in healthy mice of different ages. We found that aging was accompanied by increased surface expression of the PMN activation markers CD11b and CD63 and by enhanced CD62L shedding (Fig. 5). The basal PMN activation levels of unstimulated PMNs increased with age, reflecting age-dependent priming of PMNs (Fig. 5A-C). Moreover, the functional responses of PMNs to subsequent FPR stimulation also increased with age (Fig. 5D-F; Table 2). In particular, increased CD63 expression may be directly linked to a hyperinflammatory state in old mice because it indicates the release of proteolytic enzymes from azurophilic granules such as elastase, which are known to contribute to inflammatory tissue damage [34, 35]. Taken together, these findings demonstrate that the basal activation state of PMNs increases with age, resulting in primed PMNs that show exaggerated activation responses in the presence of inflammatory stimuli such as FPR ligands [36].

**Table 2:** Priming and activation in response to FPR stimulation are significantly greater in old mice than in young mice

	Young (< 30 weeks)	Old (≥ 30 weeks)	*p* value
Age (weeks)	15 ± 1	49 ± 2	0.00000
Base CD11b (MFI)	13,019 ± 839	16,081 ± 1,155	0.02180
Base CD11b (% pos.)	112 ± 2	17 ± 3	0.06179
Stim. CD11b (MFI)	91,840 ± 4,857	115,510 ± 5,048	0.00143
Stim. CD11b (% pos.)	83 ± 3	88 ± 2	0.08523
Base CD62L (MFI)	14,284 ± 1,032	12,305 ± 643	0.10390
Base CD62L (% pos.)	95 ± 2	96 ± 1	0.36493
Stim. CD62L (MFI)	2,137 ± 317	3,066 ± 264	0.02148
Stim. CD62L (% pos.)	37 ± 4	54 ± 3	0.00163
Base CD63 (MFI)	319 ± 31	555 ± 66	0.00019
Base CD63 (% pos.)	14 ± 1	27 ± 3	0.00003
Stim. CD63 (MFI)	1,002 ± 135	2,880 ± 329	0.00000
Stim. CD63 (% pos.)	35 ± 4	75 ± 6	0.00000
Base DHR (MFI)	50,206 ± 2 832	56,041 ± 2,000	0.05815
Phagocytosis (MFI)	34,115 ± 3,261	15,422 ± 1,611	0.00000
Phagocytosis (% pos.)	57 ± 2	35 ± 3	0.00000

The activation states of PMNs in young (< 30 weeks, *n* = 10–67) and old mice (≥ 30 weeks, *n* = 12–25) before (basal levels; Base) and after stimulation with W-peptide (stimulated levels; Stim.) were assessed with flow cytometry. The data are shown as the means ± SEMs, and the data obtained from old and young mice were compared via unpaired t tests. MFI: mean fluorescence intensity

### PMN phagocytosis decreases with age

**Fig. 6:**
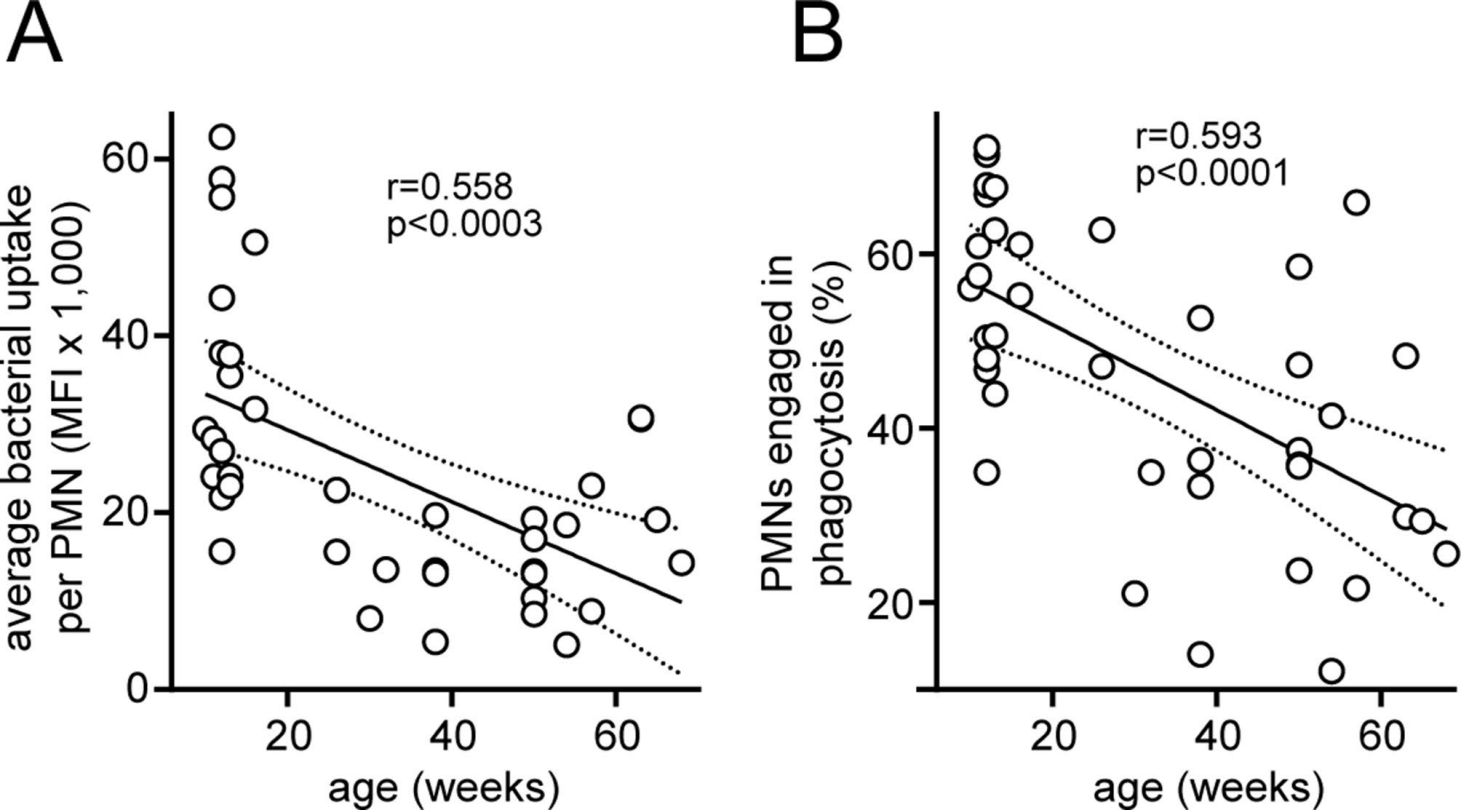
PMN phagocytosis decreases with age. The phagocytic activity of PMNs from mice of different ages was assessed with fluorescent *E. coli* particles and flow cytometry. The average number of bacterial particles taken up by PMNs (**A**) and the percentage of PMNs engaged in bacterial phagocytosis (**B**) were compared with the age of the animals studied. Linear correlations with 95% confidence intervals are shown along with the corresponding correlation coefficients and significant p values. MFI: mean fluorescence intensity

Autocrine purinergic signaling orchestrates PMN chemotaxis, which is a prerequisite for phagocytosis and the elimination of invading microorganisms [16]. Elevated extracellular ATP levels disrupt PMN chemotaxis because they interfere with the P2Y2 receptor signaling mechanisms that coordinate gradient sensing, cell polarization, and effective forward movement of PMNs during chemotaxis [16, 17, 37, 38]. Because we found that plasma ATP levels increase with age, we examined whether aging results in impaired PMN phagocytosis. Using blood samples from mice of different ages, we found that the phagocytic capacity of PMNs rapidly decreased with age (Fig. 6). The ability of PMNs to take up bacteria (Fig. 6A) as well as the percentage of PMNs engaged in phagocytosis (Fig. 6B) decreased significantly with increasing age. This decline in phagocytosis, together with the findings described above, supports the concept that extracellular ATP accumulation in response to aging contributes to PMN dysfunction and lethal bacterial infections [18, 19, 30].

### Plasma ATP and adenosine levels correlate with the priming of PMNs

**Fig. 7:**
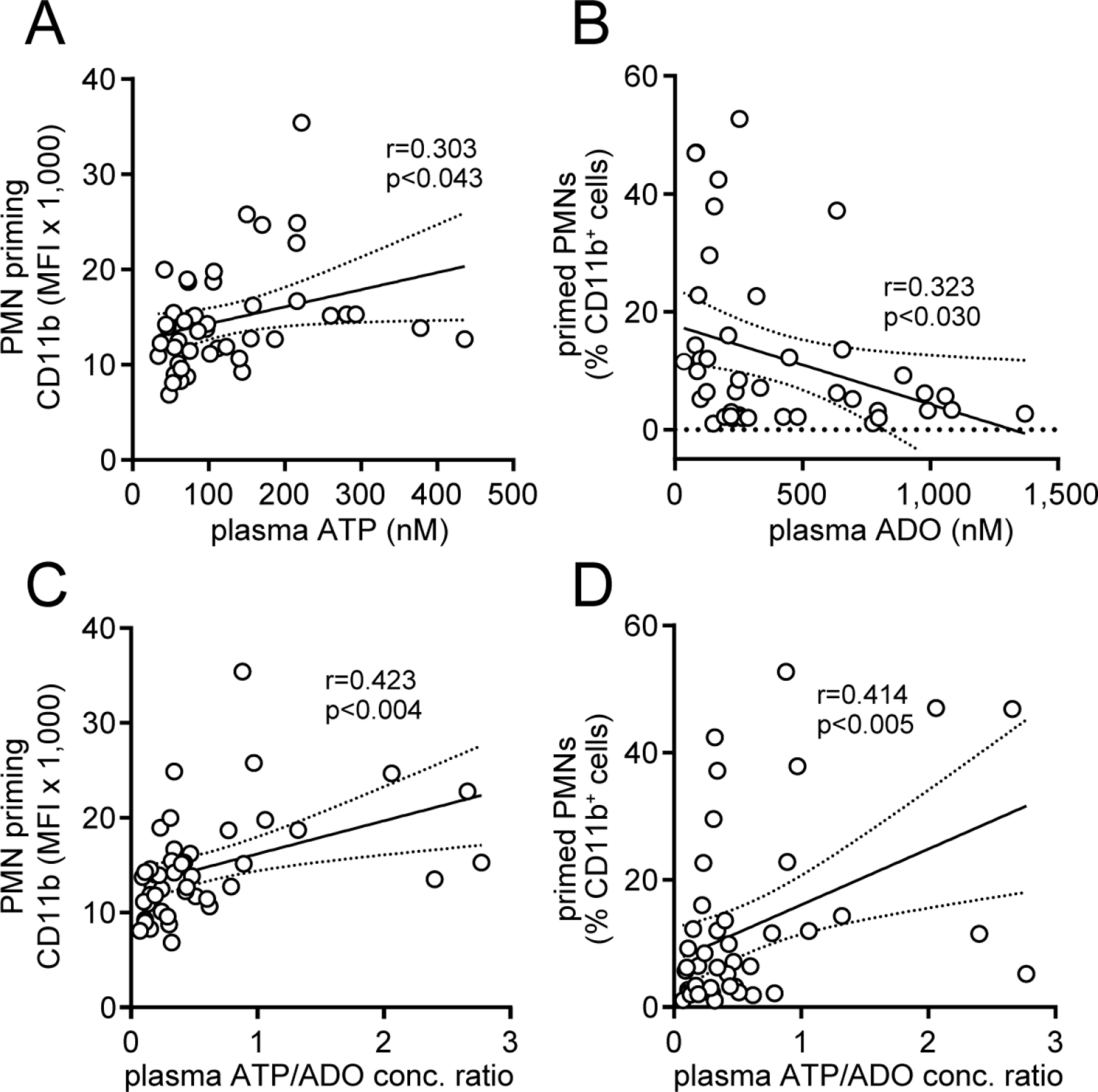
Plasma ATP and adenosine concentrations correlate with the PMN activation state in mice of different ages. The priming state of PMNs in blood samples obtained from mice of different ages was assessed by measuring CD11b expression via flow cytometry. PMN priming levels were compared with corresponding plasma ATP concentrations (**A**) and with plasma adenosine (ADO) levels (**B**). PMN priming states were also compared with the ratios of ATP and adenosine plasma concentrations (**C-D**). Linear correlations with 95% confidence intervals are shown along with the corresponding correlation coefficients and significant p values. MFI: mean fluorescence intensity

We found that the priming state of PMNs increased with the age of mice. Plasma ATP levels also increased with age, and extracellular ATP dose-dependently enhanced PMN activation. These findings suggest that age-dependent increases in plasma ATP levels contribute to PMN priming in older mice. In support of this concept, we found that the priming state of PMNs was positively correlated with the ATP concentrations measured in plasma samples from mice of different ages (Fig. 7A). A possible reason for the increase in plasma ATP levels in old mice is reduced hydrolysis of extracellular ATP to its metabolite adenosine. Therefore, we studied the relationship between plasma adenosine levels and the priming state of PMNs. We found that PMN priming levels decreased with increasing plasma adenosine levels (Fig. 7B). These findings suggest that both ATP and adenosine in mouse plasma define the priming state of their PMNs. This notion is further supported by the positive correlations we found between the ratios of the ATP and adenosine plasma concentrations and the PMN priming states in mice (Fig. 7C-D).

### Blood ATP levels increase because of reduced ATPase activity in the plasma of old mice

**Fig. 8:**
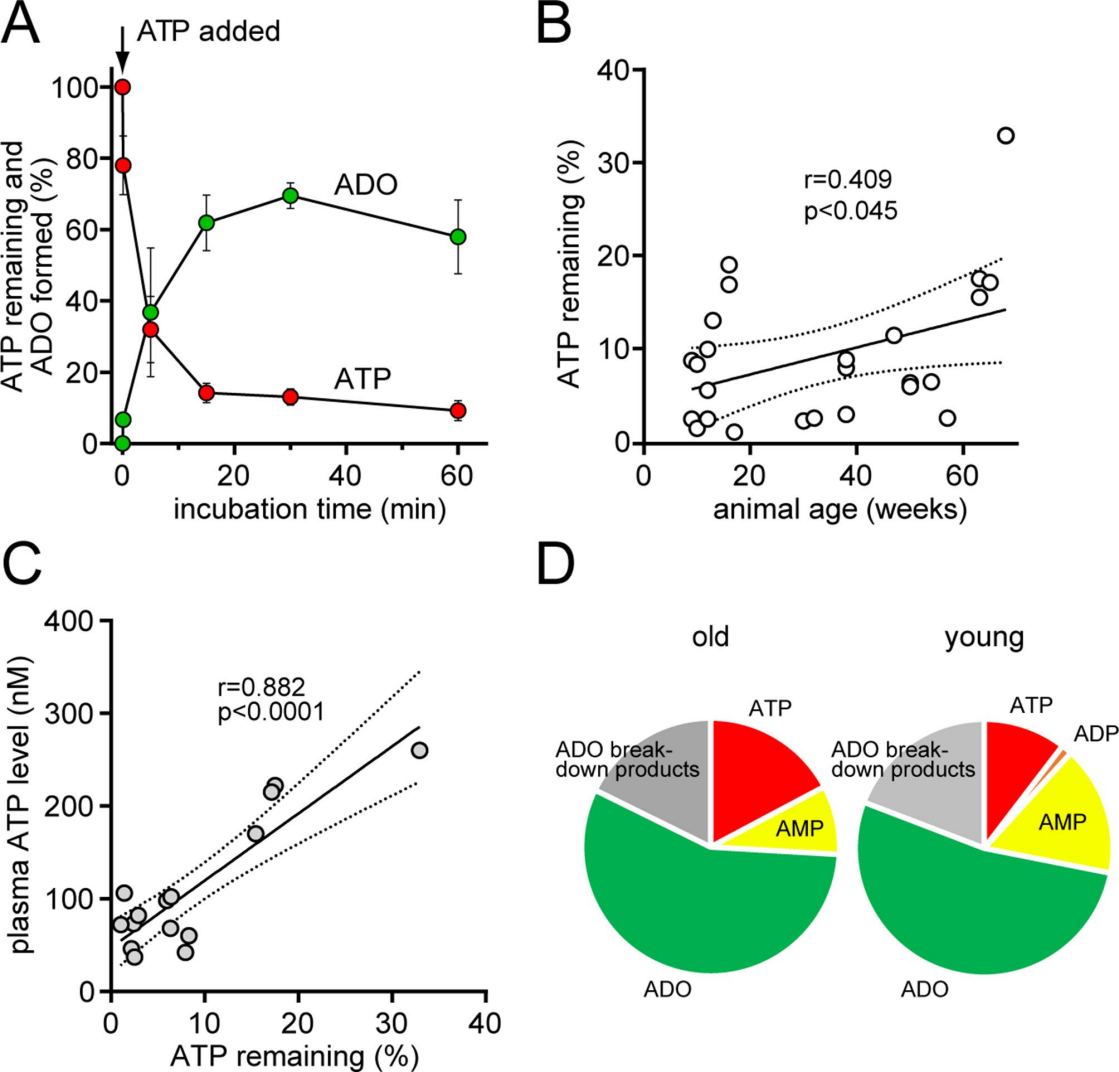
Plasma ATPase activity decreases with age. Mouse plasma was diluted 1:4 with HBSS and warmed to 37 °C; then, ATP (2 µM) was added, and ATP breakdown was analyzed. The plasma of young mice (14–16 weeks; *n* = 4) was hydrolyzed primarily to adenosine (ADO) within a 20-min incubation period (**A**). Plasma ATPase activity was estimated by determining the concentration of ATP that remained after a 10-min incubation period (**B**). ATPase activity levels in plasma samples were compared with the circulating ATP concentrations measured in plasma samples from the corresponding mice (**C**). The breakdown products formed after incubation of ATP in the plasma of young mice (≤ 20 weeks; *n* = 30) were compared with those formed by the plasma of old animals (> 60 weeks; *n* = 8) (**D**). Linear correlations with 95% confidence intervals are shown along with the corresponding correlation coefficients and significant p values in panels B and C

The findings above suggest that ATP accumulates in the plasma of old mice because ATP is not efficiently hydrolyzed in the blood. Therefore, we examined the activity of plasma ATPases in heparinized blood samples drawn from mice of different ages. ATP added to the plasma samples of young mice (< 20 weeks) was rapidly hydrolyzed, resulting in the generation primarily of adenosine (Fig. 8A). Plasma samples obtained from older mice were less capable of hydrolyzing ATP, indicating that plasma ATPase activity decreased with increasing age (Fig. 8B). The percentage of added ATP that remained unhydrolyzed in plasma samples increased with age and closely matched the circulating ATP levels found in the blood of the corresponding animals (Fig. 8C). In general, the ATP breakdown patterns generated in the plasma of old mice differed from those of young mice (Fig. 8D). Overall, these findings demonstrate that changes in the activity of plasma enzymes that hydrolyze ATP are responsible for the increased circulating ATP levels found in old mice.

### Breakdown of extracellular ATP requires divalent metal ions

**Fig. 9:**
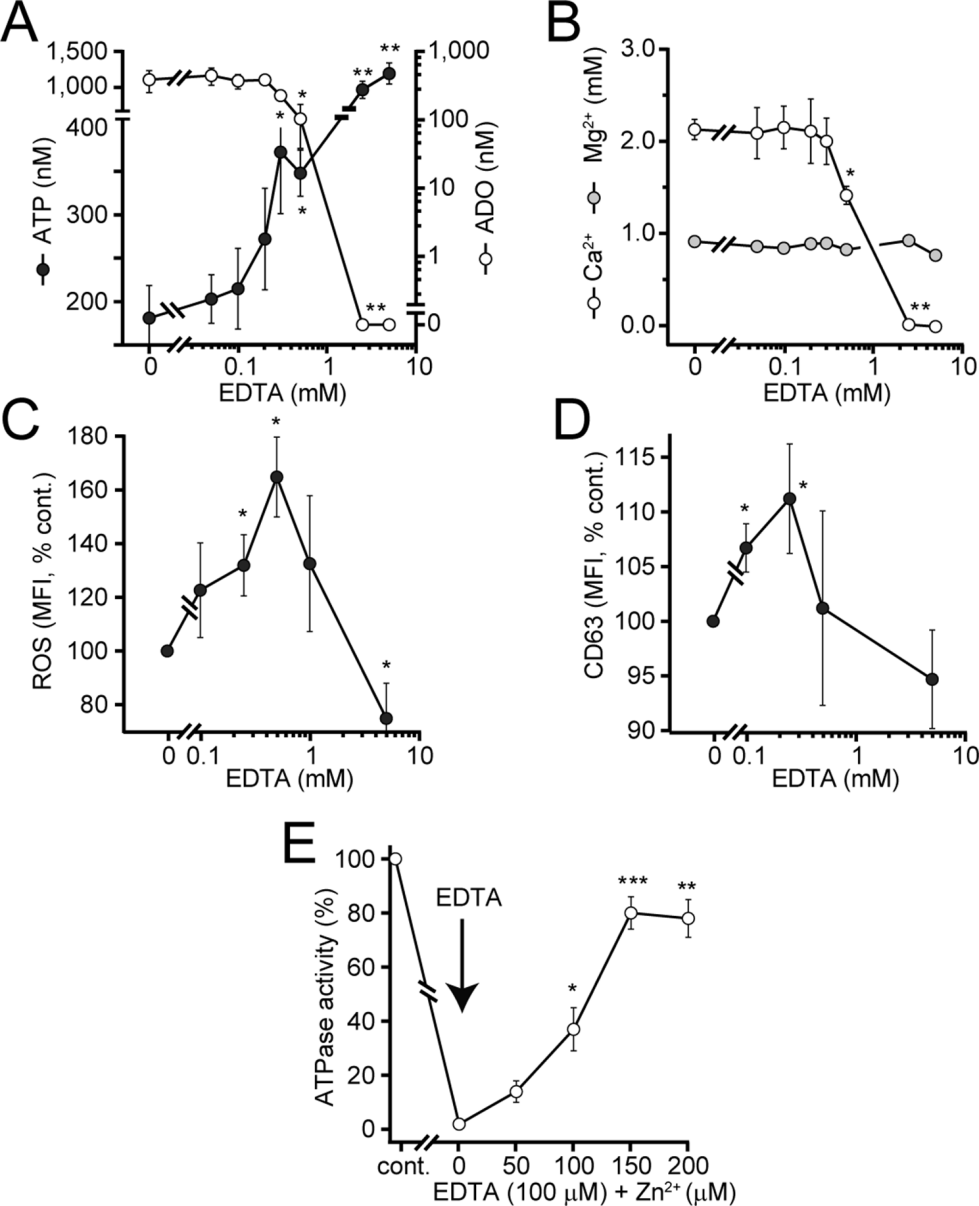
Chelation of metal ions prevents ATP hydrolysis and promotes PMN activation. Heparinized human whole blood samples (*n* = 3) were incubated at 37 °C with increasing concentrations of EDTA, and plasma ATP and adenosine levels were assessed 2 h later (**A**). The effect of EDTA on Ca^2+^ and Mg^2+^ concentrations in blood samples was determined after EDTA treatment as described in panel **A** (**B**). The effects of EDTA on FPR-induced PMN responses were assessed by treating human blood samples (*n* = 7) as described in panel **A**, stimulating cells with 50 nM fMLP (**C**) or 15 nM fMLP (**D**), and measuring the formation of ROS and the expression of the activation marker CD63 via flow cytometry (**C**-**D**). The role of Zn^2+^ ions in maintaining the ATPase activity of mouse plasma was determined by depleting diluted plasma samples (*n* = 3) of divalent ions by addition of 0.1 mM EDTA, titrating back Zn^2+^ ions at the concentrations shown, and assessing ATP breakdown capacity of these samples with a luciferase assay (**E**). The data shown are the means ± SEMs, and the data were compared with the control values (0 mM EDTA) using one-way ANOVA and Holm-Sidak post hoc test; *p < 0.05, **p < 0.01, ***p < 0.001. MFI: mean fluorescence intensity

The identities of the enzymes responsible for the breakdown of ATP in plasma are not well defined [39, 40]. However, soluble forms of ectonucleoside triphosphate diphosphohydrolases (ENTPDs), ectonucleotide pyrophosphatase/phosphodiesterases (ENPPs), ecto-5’-nucleotidase (NT5E; CD73), and alkaline phosphatase isoforms have been reported [41, 42]. These enzymes require divalent metal ions, including Ca^2+^, Mg^2+^, and Zn^2+^, which act as cofactors at their active sites or as structural elements that maintain molecular architecture [39, 43]. Therefore, the loss of divalent metal ions could explain the accumulation of extracellular ATP in response to aging. We added the metal chelator ethylenediaminetetraacetic acid (EDTA) to human whole blood as a model to examine the roles of divalent metal ions in the conversion of plasma ATP and the regulation of PMN functions. EDTA added to whole blood resulted in a dose-dependent accumulation of extracellular ATP, which was paralleled by a decrease in plasma adenosine levels (Fig. 9A). These results demonstrate that divalent metal ions are needed to convert extracellular ATP to adenosine, which can be internalized by cells. EDTA concentrations as low as 0.5 mM had a significant impact on ATP hydrolysis in human blood. Interestingly, equivalent EDTA concentrations had little to no effect on the corresponding plasma Ca^2+^ and Mg^2+^ levels (Fig. 9B). Removal of divalent ions with EDTA not only blocked ATP breakdown but also increased the activation of ROS production (Fig. 9C), CD63 expression (Fig. 9D), and CD11b and CD66b expression (data not shown) in response to PMN stimulation. EDTA concentrations of up to 0.5 mM enhanced those PMN responses, while higher EDTA concentrations caused suppression, likely due to complete chelation of free Ca^2+^ ions (see Fig. 9B) that are indispensable for cell activation. Addition of Zn^2+^ to mouse plasma that had been depleted of divalent ions with EDTA dose-dependently recovered ATPase activity (Fig. 9E). These findings support the concept that Zn^2+^ has a central role as a co-enzyme that helps control ATP levels in mouse plasma. Taken together, these findings suggest that metal ions such as Ca^2+^, Mg^2+^, and particularly Zn^2+^ play important roles in the regulation of extracellular ATP and adenosine levels in mouse and human blood.

## Discussion

PMN dysfunction leads to a wide range of inflammatory and infectious diseases that afflict primarily older patients [6, 44]. As the predominant innate immune cell population, PMNs play critical roles in host defense. A clearer understanding of the underlying mechanisms by which aging impairs PMN functions could have a major impact on the health care of older adults. PMNs are equipped with a highly sensitive system to detect the presence of invading microorganisms. This system comprises signal amplification steps that involve mitochondrial translocation and ATP production, localized ATP release through pannexin 1 channels, and autocrine stimulation of P2Y2 and A2a purinergic receptors [13, 17, 37]. These processes control cell polarization, chemotaxis, priming, activation, and the phagocytosis and elimination of microbes by PMNs. However, these delicate mechanisms are not impervious to external interference. Specifically, the accumulation of excessive concentrations of ATP and its hydrolysis product adenosine in the extracellular milieu leads to improper stimulation of the purinergic receptors of PMNs, which impairs host defense functions and promotes inflammatory complications [17, 18, 45].

We show here that the ATP concentration in the plasma of mice increases with age. This increase in ATP levels was paralleled by a decrease in the ability of plasma enzymes to generate adenosine and other ATP breakdown products. These are novel results with potential clinical importance because reduced plasma adenosine levels have been described in older humans [46]. In children, the activity levels of plasma enzymes that can hydrolyze ATP and its breakdown products have been reported to change with age [47]. Interestingly, the activities of these plasma ectoenzymes were found to be greater in infants and young children than in adolescents [47, 48]. Taken together with our current results, these findings suggest that the mechanisms that regulate plasma adenylate levels change continuously throughout the aging process. Most enzymes capable of converting extracellular ATP and adenosine are bound to cell surfaces. Therefore, changes in the expression of these cell-bound ectoenzymes may contribute to altered adenylate levels in response to aging. However, little is known about how the activity and expression levels of these ectoenzymes change with age [49].

The results of our study demonstrate that soluble enzymes in plasma play major roles in determining circulating ATP concentrations in mice and humans. Soluble plasma ATPases may thus provide a necessary buffer that isolates the purinergic signaling systems of PMNs from interference by ATP that is released in response to tissue damage and inflammation. This notion is supported by previous work showing that inappropriate accumulation of systemic ATP under inflammatory or infectious conditions has detrimental effects on PMN functions [18, 19, 30]. In accordance with this concept, we found here that decreased plasma ATPase activity in old mice was paralleled by impaired bacterial phagocytosis, increased PMN priming, and an inflammatory cascade that culminated in lethal sepsis following bacterial infection.

Priming of PMNs can cause excessive PMN activation in response to subsequent cell stimulation. We found that the primed PMNs of old mice responded significantly more vigorously to FPR stimulation than the PMNs of younger mice. We used W-peptide to stimulate mouse PMNs, which is an agonist acting on murine FPR1 and FPR2 subtypes [50]. While W-peptide can model inflammatory responses associated with FPR1 signaling in humans, differences between murine and human PMNs and their respective FPR subtypes exist, and these differences must be considered when attempting to translate our results to the human scenario. Using W-peptide, we found that the degranulation of azurophilic granules by the PMNs of old mice was significantly more pronounced than that of cells from young mice. Azurophilic granules contain a number of powerful proteases, such as neutrophil elastase, that can inflict major collateral damage to host tissues [34, 35]. Indeed, old mice developed profound inflammatory complications and lethal sepsis in response to bacterial infection, while young mice fared significantly better when subjected to the same infectious challenge. In contrast to their older counterparts, young mice were able to control the systemic spread of bacteria following intra-abdominal infection and they showed little, if any, evidence of inflammatory PMN responses.

Our data point to systemic ATP as a major cause of these differences between old and young mice, apparently because ATP accumulation triggers excessive P2Y2 receptors activation of PMNs. However, ATP has also been shown to modulate apoptosis of different cell types including PMNs [51, 52]. Apoptosis is mediated by P2X7 receptors that require ATP concentrations in the high micromolar to millimolar range, which is beyond the ATP levels we found in the plasma of old mice. Thus, ATP-mediated apoptosis of PMNs is an unlikely contributor to the impaired immune responses we saw in old mice.

While our current study focused on PMNs, other immune cell subsets also contribute to a successful antimicrobial host defense. Among these cells are macrophages. Like PMNs, macrophages depend on autocrine signaling via P2Y2, A2, and other purinergic receptors to regulate chemotaxis, NLRP3 inflammasome activation, and other cell functions [53–55]. Systemic ATP accumulation could interfere with these purinergic signaling mechanisms and thereby disrupt macrophage functions such as phagocytosis, cytokine secretion, and ROS production that were reported to deteriorate in response to aging [56]. Further studies are needed to examine the exact mechanisms by which ATP accumulation and aging impact PMNs, macrophages, and other immune cell subsets.

Nevertheless, the critical role of plasma ATP accumulation as a cause of PMN dysregulation is supported by our current findings that the addition of exogenous ATP to blood samples of healthy young mice replicated the dysfunctional PMN responses observed in old mice. Taken together, our study indicates that systemic ATP accumulation and decreased ATPase activity in plasma are major causes of PMN dysfunction in response to aging. Although several soluble plasma enzymes that can convert extracellular ATP and its breakdown products have been identified, their regulation has not been fully elucidated [41, 43, 57]. However, all these enzymes require divalent metal ions as cofactors for nucleotide dephosphorylation [58, 59]. ENTPD isoforms such as CD39, ENPP isoforms, ecto-5′-nucleotidase (CD73), alkaline phosphatases, and adenosine deaminases depend on Ca^2+^, Mg^2+^, or Zn^2+^ as coenzymes or structural elements [43, 57]. Using blood samples from healthy humans, we found that decreasing these divalent metal ions by chelation with EDTA could replicate the effects of aging on extracellular ATP levels and PMN functions. These findings suggest that reduced levels of these metal ions contribute to PMN dysfunction in response to aging. Deficiencies in Zn^2+^, Ca^2+^, and other micronutrients are well-known issues in elderly subjects [60], which can contribute to immune dysregulation, reduced clearance of extracellular ATP, and inflammatory PMN responses [40, 60–64]. Clinical studies have shown that Zn^2+^ supplementation can significantly reduce the risk of infections in elderly individuals [61, 62, 65]. We found that Zn^2+^ is essential for the activity of plasma ATPases in mice. Together with the evidence described above, this observation warrants further studies to investigate whether deficiencies in Zn^2+^ or other divalent metal ions cause systemic ATP accumulation and immune dysfunction in mice as well as in humans. The results of such studies will be essential for the development of novel therapeutical strategies that counteract the deterioration of cellular immune responses in older individuals.

## Conclusions

Our findings confirm previous studies that have shown that PMN functions deteriorate with age. However, our work provides novel insights into the fragmented understanding of the underlying causes. Specifically, our results suggest that the well-described deleterious consequences of Zn^2+^ deficiency in elderly patients may be linked to impaired ATPase activity that dysregulates PMN functions because of the accumulation of ATP in the plasma. Further work is needed to establish the causal relationships between Zn^2+^, other divalent metal ions, plasma ATP levels, and PMN dysfunction in older humans and in patients. We hope that our current study will inspire such work and help with the development of novel therapeutic strategies that reduce the risk of infections and inflammatory disorders in the aging population.

## Data Availability

No datasets were generated or analysed during the current study.
